# Structural insight into *Escherichia coli* CsgA amyloid fibril assembly

**DOI:** 10.1128/mbio.00419-24

**Published:** 2024-03-19

**Authors:** Fan Bu, Derek R. Dee, Bin Liu

**Affiliations:** 1 The Hormel Institute, University of Minnesota, Austin, Minnesota, USA; 2 Faculty of Land and Food Systems, The University of British Columbia, Vancouver, British Columbia, Canada; 3 Department of Pharmacology, University of Minnesota Medical School, Minneapolis, Minnesota, USA; University of Michigan—Ann Arbor, Ann Arbor, Michigan, USA

**Keywords:** functional amyloids, protein aggregation, curli, electron microscopy, biofilms, bacteria, protein structure-function

## Abstract

The discovery of functional amyloids in bacteria dates back several decades, and our understanding of the *Escherichia coli* curli biogenesis system has gradually expanded over time. However, due to its high aggregation propensity and intrinsically disordered nature, CsgA, the main structural component of curli fibrils, has eluded comprehensive structural characterization. Recent advancements in cryo-electron microscopy (cryo-EM) offer a promising tool to achieve high-resolution structural insights into *E. coli* CsgA fibrils. In this study, we outline an approach to addressing the colloidal instability challenges associated with CsgA, achieved through engineering and electrostatic repulsion. Then, we present the cryo-EM structure of CsgA fibrils at 3.62 Å resolution. This structure provides new insights into the cross-β structure of *E. coli* CsgA. Additionally, our study identifies two distinct spatial arrangements within several CsgA fibrils, a 2-CsgA-fibril pair and a 3-CsgA-fibril bundle, shedding light on the intricate hierarchy of the biofilm extracellular matrix and laying the foundation for precise manipulation of CsgA-derived biomaterials.

**IMPORTANCE:**

The visualization of the architecture of *Escherichia coli* CsgA amyloid fibril has been a longstanding research question, for which a high-resolution structure is still unavailable. CsgA serves as a major subunit of curli, the primary component of the extracellular matrix generated by bacteria. The support provided by this extracellular matrix enables bacterial biofilms to resist antibiotic treatment, significantly impacting human health. CsgA has been identified in members of *Enterobacteriaceae*, with pathogenic *E. coli* being the most well-known model system. Our novel insights into the structure of *E. coli* CsgA protofilaments form the basis for drug design targeting diseases associated with biofilms. Additionally, CsgA is widely researched in biomaterials due to its self-assembly characteristics. The resolved spatial arrangements of CsgA amyloids revealed in our study will further enhance the precision design of functional biomaterials. Therefore, our study uniquely contributes to the understanding of CsgA amyloids for both microbiology and material science.

## INTRODUCTION

The traditional structure-function paradigm has played a central role in structural biology for many decades, yet approximately 30% of eukaryotic proteins are intrinsically disordered proteins (IDPs) or contain intrinsically disordered regions (IDRs) (1), where they do not fold into well-defined 3D structures to carry out functions. Ubiquitous unfolded structures of IDPs/IDRs enable functional versatility (2), encompassing signaling, intercellular transport, and transcriptional regulation (3, 4). Their frequent hydrogen bond disruptions due to conformational changes, on the other hand, render IDPs sensitive to environmental fluctuations and are prone to aggregation (4, 5). IDPs are often associated with pathological amyloids like α-synuclein and Aβ, which represent hallmarks of Parkinson’s disease and Alzheimer’s disease, respectively (6). However, a category of IDPs known as functional amyloids has been discovered to play diverse physiological roles, including chemical storage, structural scaffolding, information carriers, loss-of-function, and gain-of-function (7, 8).

Curli, a prototypical functional amyloid formed by IDPs, serves a pivotal role in bacteria. Curli fibrils, widely expressed by Enteric bacteria like *Escherichia coli* and *Salmonella* spp., integrate into the extracellular matrix, promoting structural scaffolding, adhesion, and host colonization (9). This enhances biofilm stability and resilience, protecting bacteria against nutritional, mechanical, chemical, and physical stresses with significant implications for human health (7). Bacteria within biofilms exhibit a distinct ability to resist antibiotic-induced eradication, leading to numerous treatment challenges encountered in clinical settings (10). The colonization of biofilms in gut mucus is also closely linked to conditions such as colorectal cancer and inflammatory bowel disease (11). Furthermore, there is evidence suggesting that curli fibrils expressed in the gastrointestinal tract have the potential to spread into the peripheral nervous system and trigger neurodegenerative diseases (12, 13). Therefore, over the past few decades, numerous studies have delved into understanding the regulation and biogenesis of curli, as well as the structure, function, and assembly of curli fibrils.

The curli field has accelerated since the successful isolation of wild-type CsgA in 2002 (14). The curli system consists of seven proteins encoded by two discrete operons (*csgBAC* and *csgDEFG*), which regulate the assembly of a major structural subunit, CsgA (15), and a minor subunit, CsgB, into curli fibrils (16). Initially, CsgA and CsgB are soluble IDPs when both are secreted through the CsgG-CsgF pore complex to the outer membrane surface of *E. coli*, where CsgA aggregation is nucleated by CsgB (17–20). Both CsgA and CsgB comprised three discrete segments: a signal peptide, an N-terminal (N22 and N23) peptide, and a C-terminal amyloidogenic region composed of five imperfect repeats (R1-R5) (21). CsgC, a chaperone located in the periplasm, prevents amyloid formation within the cell (22, 23), while CsgD is a transcriptional protein that regulates *csgBAC* operons. Accessory factors CsgE and CsgF bind to the CsgG channel, aiding in the secretion of CsgA and CsgB and curli amyloid fibril formation (24–28).

Several approaches have been employed in previous studies to gain structural insights into both soluble CsgA and CsgA fibrils, the major component of the curli system. Molecular simulation studies revealed that predicted CsgA structures with beta-helical conformations exhibited the lowest root mean square deviation (RMSD), while the role of N22 remains debatable (29–32). Some structure prediction servers suggest that N22 adopts a β-sheet structure, while others indicate it remains disordered (30). Additionally, it was predicted that CsgA structures could adopt both left-hand and right-hand chiralities (29). Solid state Nuclear magnetic resonance (NMR) and X-ray diffraction were also used to gain insights into the structure of *E. coli* CsgA fibrils (21, 33), suggesting that β-helices rather than parallel β-sheets form the structure. Interestingly, several peptide segments of CsgA were discovered to adopt a canonical steric zipper architecture (21). Nevertheless, obtaining a high-resolution structure of CsgA fibrils remains challenging, which is attributed to the high propensity of aggregation and the intrinsically disordered nature of CsgA. The lack of comprehensive structural information has not only impeded a deeper understanding of CsgA within a complex matrix and its potential for cross-seeding with other human proteins but also has hindered the rational design of drugs and biomaterials (34).

**Fig 1:**
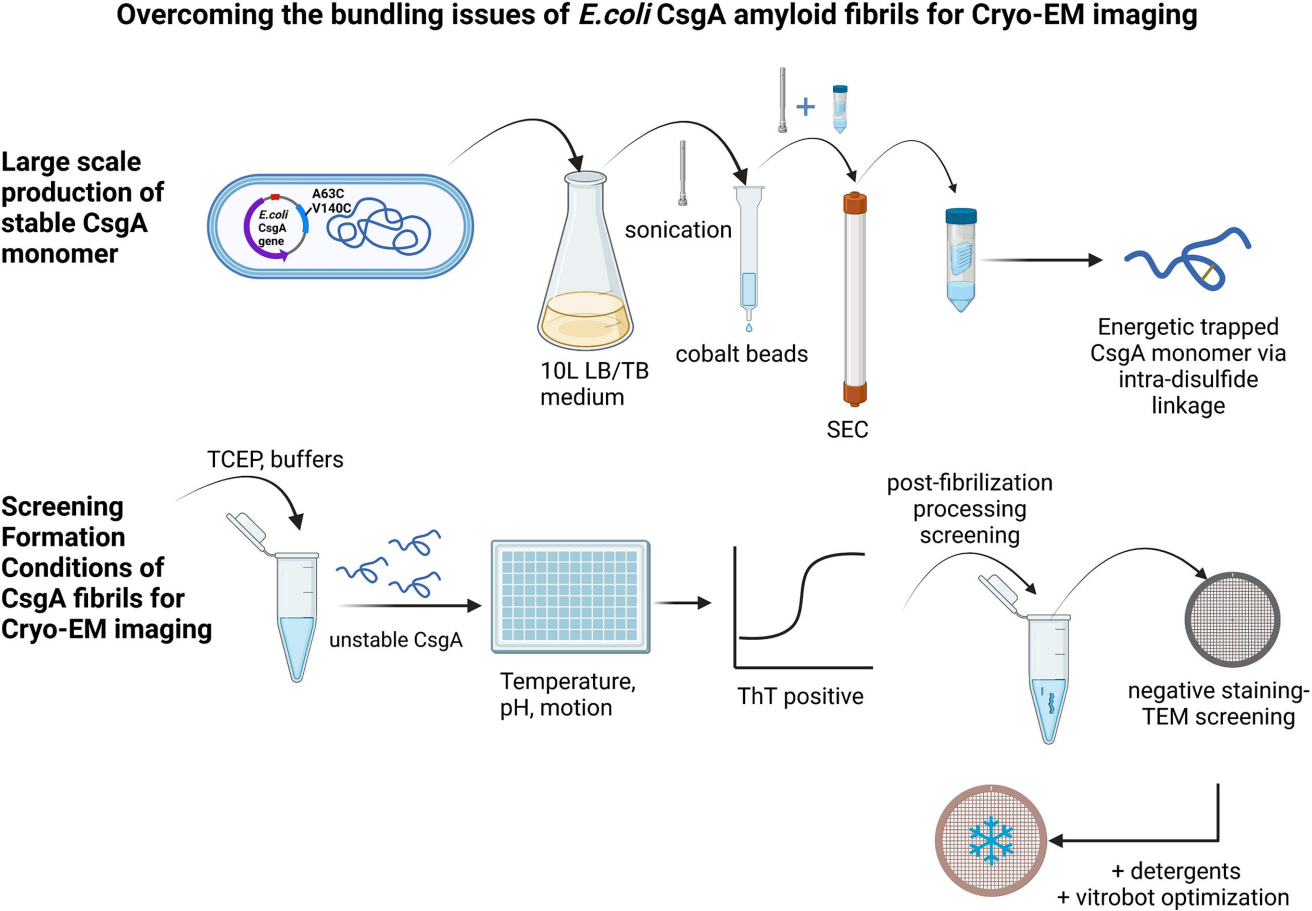
Schematic of how to overcome the bundling issues of *E. coli* CsgA fibrils for cryo-EM data collection. The optimization process involved adjusting the medium type, induction temperature, and induction time to achieve the highest yield of CsgA monomer. Then, various conditions during fibrillation (pH, salt, agitation, and additives) and de-bundling methods (heat, detergent, sonication, and buffer exchange) after assembly were tested to obtain de-bundled CsgA fibrils suitable for cryo-EM imaging. Lastly, data processing procedures were fine-tuned to generate good 2D classes and 3D density maps.

The most recent attempt to determine the structure of *E. coli* CsgA fibrils through cryo-electron microscopy (cryo-EM) was unsuccessful, because of the high propensity of these fibrils to bundle, which is undesirable for cryo-EM data processing (35). Sleutel et al. (35) suggested that the *E. coli* CsgA might adopt irregular or even systematic higher-order structures. In our study, to avoid the bundling issues of CsgA and to solve the structure of *E. coli* CsgA fibrils, we have developed a novel approach (Fig. 1). First, we chose to use the disulfide-engineered CsgA (36), then focused on maximizing the yield and purity of CsgA monomers for fibrillation, followed by optimizing the fibrillation conditions to enhance electrostatic repulsion and de-bundle the CsgA fibrils. Finally, we attempted cryo-EM imaging processes with different box sizes to obtain high-quality 2D classes. For the first time, we have uncovered the structure of *E. coli* CsgA fibrils at 3.62 Å resolution and identified two distinct spatial organizations among several CsgA fibrils.

## RESULTS AND DISCUSSION

### Optimizing the expression and purification of CsgA

The purification process for wild-type CsgA typically involves denaturing conditions (37) (using 8 M guanidine hydrochloride) to preserve its monomeric state, as it readily forms amyloid fibrils within a few hours under non-denaturing conditions. However, the limited yield of wild-type CsgA poses a challenge in obtaining enough monomeric CsgA for screening various fibrillation conditions, which is essential for obtaining desired fibrils for cryo-EM analysis. We chose an engineered CsgA with two designed cysteines (A63C and V140C), developed by Balistreri et al. (36), as this selection offers several advantages for structural analysis. Firstly, it increased the expression yield of CsgA monomer sufficiently for screening under various conditions. Secondly, this engineered CsgA remains soluble and monomeric for approximately 2 months. The aggregation process can be easily initiated by introducing reducing agents, and the manipulation of aggregation is not interfered with by oligomers formed prior to adding the reducing agent. Thirdly, these mutations were found to not affect the CsgA fibril morphology (36), as the mutated residues are non-gatekeeper residues (38). During the purification of this CsgA, the expression level was still relatively low although higher than the wild-type CsgA, as no discernible band appeared in the SDS-PAGE gel following Isopropyl-β-D-Thiogalactopyranoside (IPTG) induction (Fig. S1A). The expression of CsgA could only be confirmed through western blotting (Fig. S1B). Purification using nickel affinity chromatography retained several impurities and, in addition, a significant loss of CsgA was found during the subsequent spin filter and size exclusion chromatography steps (Fig. S1C and D). Interestingly, we found that using affinity chromatography with cobalt rather than nickel beads resulted in a relatively purer CsgA, along with an increased yield (Fig. S1E and F). Additionally, we observed that replacing Luria-Bertani (LB) with Terrific Broth (TB) medium increased the expression of CsgA, while inducing a bulk culture (>1 L) at a lower temperature (28°C vs 37°C), rather than conducting several small-scale cultures, had a positive effect on the expression level. The induction length at 37°C, on the other hand, had a negligible impact on the yield (Fig. S1B). Finally, the production of CsgA monomers was optimized using the aforementioned conditions, resulting in a substantial increase in yield from approximately 0.8 mg/L to about 2 mg/L.

### Optimizing the fibrilization conditions of CsgA for cryo-EM imaging

Various screening conditions have been observed to significantly influence the morphology of pathological and functional amyloid (39). Identifying an optimal condition that results in amyloid suitable for data processing is a crucial determinant in the successful construction of the final cryo-EM model. While *in vitro* screening conditions may not fully represent the native structure of amyloids, the *in vitro* amyloid structures solved so far have indeed provided valuable insights into the physiological form (40, 41). It would be exceptionally valuable for *E. coli* CsgA amyloid fibrils, as no cryo-EM structures have been reported previously. Therefore, to acquire CsgA fibrils suitable for cryo-EM imaging, we assessed both the fibrillation kinetics and fibril morphology of CsgA. Negative-staining transmission electron microscopy (TEM) images were obtained for CsgA fibrils formed under all screening conditions regardless of the ThT signals because ThT kinetics are known to be influenced by buffer conditions and do not always accurately indicate the formation of fibrils. Our screening involved various factors such as pH, buffer composition, detergent presence, agitation, and temperature. The initial fibrillation was performed in Phosphate buffered saline (PBS) and Tri-HCl buffer (pH 7.2). As expected, CsgA fibrils are formed from CsgA monomer (Fig. S1G) into a cluster of elongated straight fibrils and curli fibrils (15, 36) (Fig. S2A and B) upon the addition of tris(2-carboxyethyl)phosphine (TCEP) (Fig. S1H). While the fibrillation of wild-type CsgA has been previously assessed under a wide range of conditions, including variations in pH (3–9), salt concentration (0–500 mM), and in the presence of seeding (20, 35), none of these conditions were successful in eliminating the bundling observed with CsgA fibrils. It is worth noting that CsgA contains only 4 basic amino acids (AAs) and 10 acidic AAs. Consequently, at any pH below its isoelectric point of 5.73, CsgA carries only four positive charges, which can potentially lead to bundling issues. Since the number of acidic AAs (10) exceeds that of basic AAs (4), placing CsgA in a basic environment can maximize electrostatic repulsion among negatively charged residues. In addition, a significant benefit of choosing engineered CsgA is that the incorporation of two engineered Cys residues introduces two additional negative charges at pH higher than 8. Therefore, N-cyclohexyl-3-aminopropanesulfonic acid buffer (CAPS) buffer (pH 10.4) was chosen to minimize the bundling issue during CsgA fibrilization, while HEPES buffer (pH 7) was chosen for comparison.

**Fig 2:**
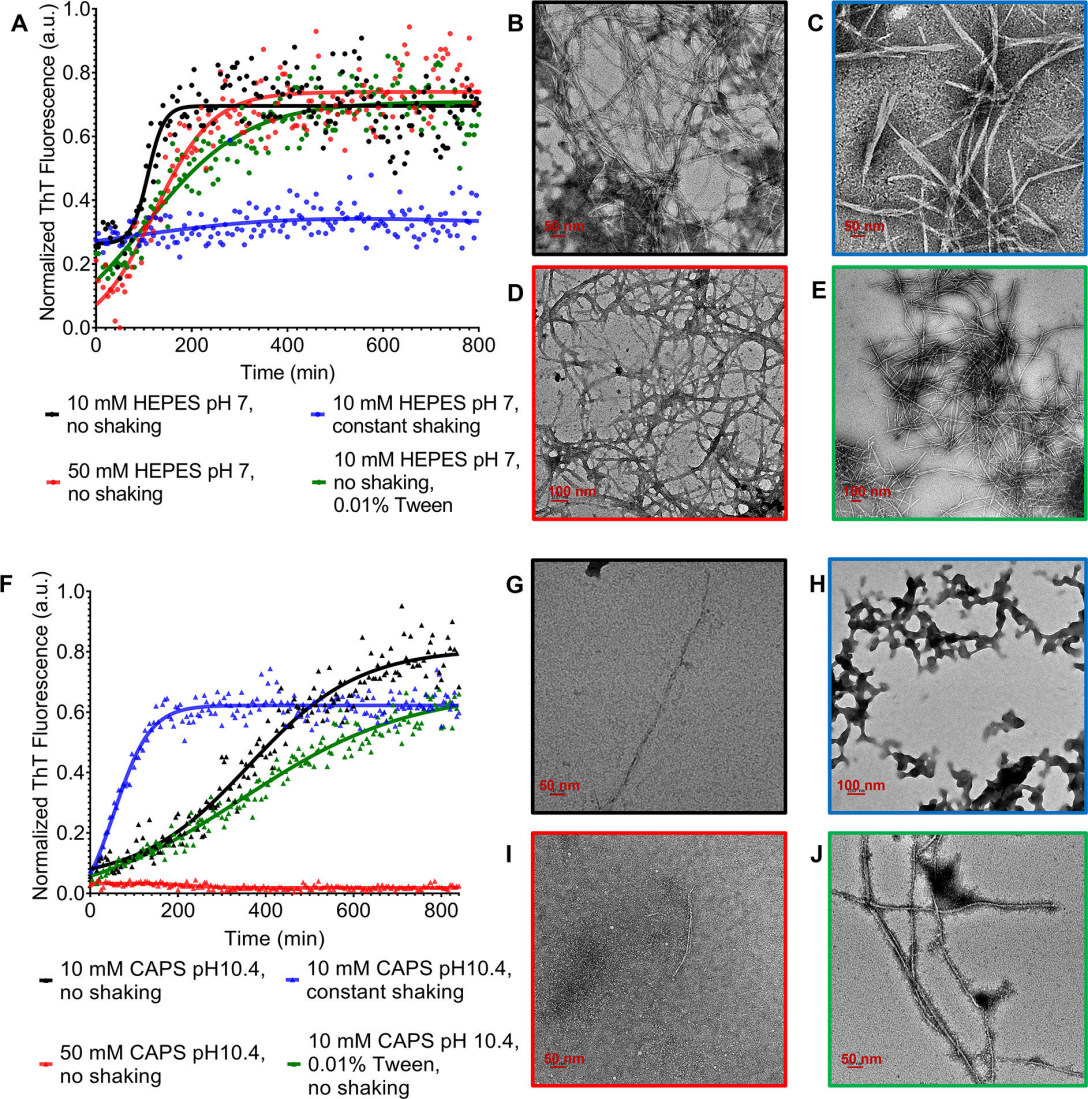
Effect of different growth conditions on CsgA fibrillation behavior. (**A**) The aggregation kinetics of CsgA in HEPES buffer were monitored by ThT fluorescence. The morphology of CsgA fibrils was characterized by negative-staining TEM. (**B**) 20 µM CsgA in 10 mM HEPES, pH 7, without shaking. (**C**) 20 µM CsgA in 10 mM HEPES, pH 7, with 200 rpm constant shaking. (**D**) 20 µM CsgA in 50 mM HEPES, pH 7, without shaking. (**E**) 20 µM CsgA in 10 mM HEPES, pH 7, without shaking + 0.01% Tween 20. (**F**) The aggregation kinetics of CsgA in CAPS buffer were monitored by ThT fluorescence. (**G**) 20 µM CsgA in 10 mM CAPS, pH 10.4, without shaking. (**H**) 20 µM CsgA in 10 mM CAPS, pH 10.4, with 200 rpm constant shaking. (**I**) 20 µM CsgA in 50 mM CAPS, pH 10.4, without shaking. (**J**) 20 µM CsgA in 10 mM CAPS, pH 10.4, without shaking + 0.01% Tween 20.

The fibrillation kinetics of CsgA in both buffers exhibited a sigmoidal time profile (Fig. 2). Notably, the lag time of CsgA in CAPS buffer (pH 10.4) was longer than in HEPES buffer (pH 7) (Fig. 2A and F). Similarly, negative-staining TEM analysis revealed a significant reduction in the tendency of CsgA to bundle in CAPS buffer (Fig. 2G through J), aligning with the proposed mechanism. In both buffers, the fibrillation kinetics of CsgA were slowed down with an increase in salt concentration. Furthermore, in 50 mM CAPS buffer, fibril formation was largely inhibited, in agreement with the negative-staining TEM (Fig. 2I). This could result from a combined effect of both high pH and high ionic strength, largely inhibiting aggregation by minimizing hydrophobic interactions. It is worth noting that the observed differences might indicate the existence of different fibril polymorphs (42–44) or be due to other effects of the various buffer conditions on the ThT kinetics and TEM staining. The addition of detergent (Tween 20) decelerated the aggregation kinetics in both buffers, resulting in straighter fibrils, which is beneficial for cryo-EM imaging (Fig. 2E and J). However, agitation led to the formation of branched and short CsgA fibrils in HEPES buffer and the formation of amorphous aggregates in CAPS buffer (Fig. 2C and H). Neither of these morphologies is beneficial to cryo-EM imaging. While some studies found that the presence of crystallization additive buffers enhanced the formation of nicely structured and well-separated fibrils (41), none of the crystallization additive reagents improved the morphology of CsgA fibrils (Fig. S3A through F). Importantly, post-fibrillation processing had a drastic effect on the morphology of CsgA fibrils (Fig. S3G through L). Heating had a de-bundling effect on the fibrils (Fig. S3G and H), whereas dialysis against water led to severe bundling of short fibrils (Fig. S3I and J). Too low of an ionic strength likely decreased the solubility of CsgA fibrils, making them colloidally unstable. Sonication after fibril formation, similar to the consequences of shaking during the fibrillation, resulted in short, branched fibrils that were not suitable for cryo-EM data processing (Fig. S3K and L).

### Cryo-EM reveals beta-helical architecture within a single CsgA fibril

**Fig 3:**
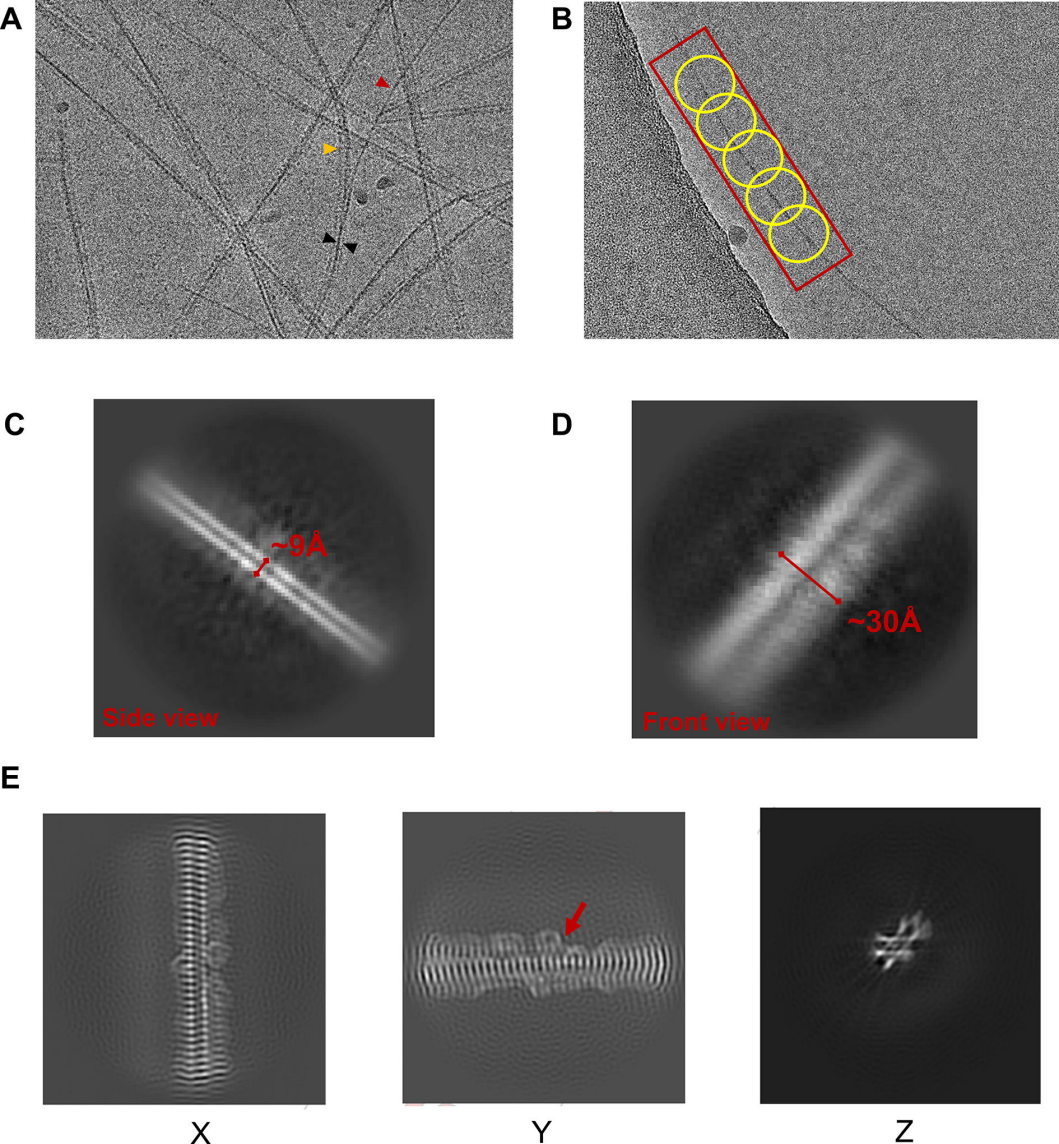
Two-dimensional representations of *E. coli* CsgA fibrils. (**A**) A representative cryo-EM image of CsgA fibrils obtained in 10 mM CAPS + 0.01% Tween 20, pH 10.4, buffer without shaking, and treated with post-fibrillation heating. The red and yellow arrows indicate a single fibril and 2-CsgA-fibril pair, respectively, derived from a 3-CsgA-fibril bundle (black arrow). (**B**) Sections of CsgA fibrils were picked using Blob picker with some overlapping between picks. Side (**C**) and front (**D**) views of a single CsgA fibril (representative 2D classes). (**E**) Central slice of the map in three orthogonal projections.

The final CsgA fibrils for cryo-EM imaging were formed in 10 mM CAPS buffer with 0.01% Tween 20, pH 10.4, without shaking, and heated at 70°C for 30 min. A cryo-EM image of CsgA fibrils (Fig. 3A) indicated that approximately three fibrils were bundled together to form a long and straight fibril. Occasionally, a single fibril was separated from the bundles of three CsgA fibrils. A blob picker with a range of particle diameters (20–120 Å) was used to pick up particles for further generating initial 3D models (Fig. 3B). We varied the box sizes in our attempt to extract CsgA particles, aiming for optimal initial models. The chosen box size corresponds to the number of included CsgA monomers (the length of the fibril) for generating 3D models. Notably, during the step of extracting particles using assigned box sizes, we found that using excessively large box sizes (320-pixel box size) resulted in poor 2D classes, likely due to the increased structural variation with the length of the fibrils. Conversely, excessively small box sizes (160-pixel box size) led to the loss of some important protofilament interactions, resulting in poor 3D density. Acceptable initial 3D models were generated by using box sizes of 218 and 260 pixels (Fig. S4 to S6; Table S1). The helical thickness (~9 Å) and helix width (~30 Å) were measured from the 2D classes of the side and front views of a single fibril, respectively (Fig. 3C and D). These measurements were consistent with the *E. coli* CsgA structure revealed by molecular simulation and X-ray diffraction data (Table S2). In contrast, *Pontibacter korlensis* R15.5, which is a homolog of CsgA (sequence identity 32.73%, Fig. S7) and belongs to a different class of curli subunits (centrosymmetric), displayed a helix thickness of approximately 20 Å (35) that differs from both predictions and our experimental data (~9 Å) obtained for *E. coli* CsgA. Additionally, a central slice of the CsgA fibril map in three orthogonal projections revealed some additional density on both sides of the fibrils, likely attributed to the flexible N22 region (Fig. 3E). Based on the density, several residues of N22 possibly interact with side chains of the fibrils, thereby resulting in the “ear-like” density surrounding the fibrils.

**Fig 4:**
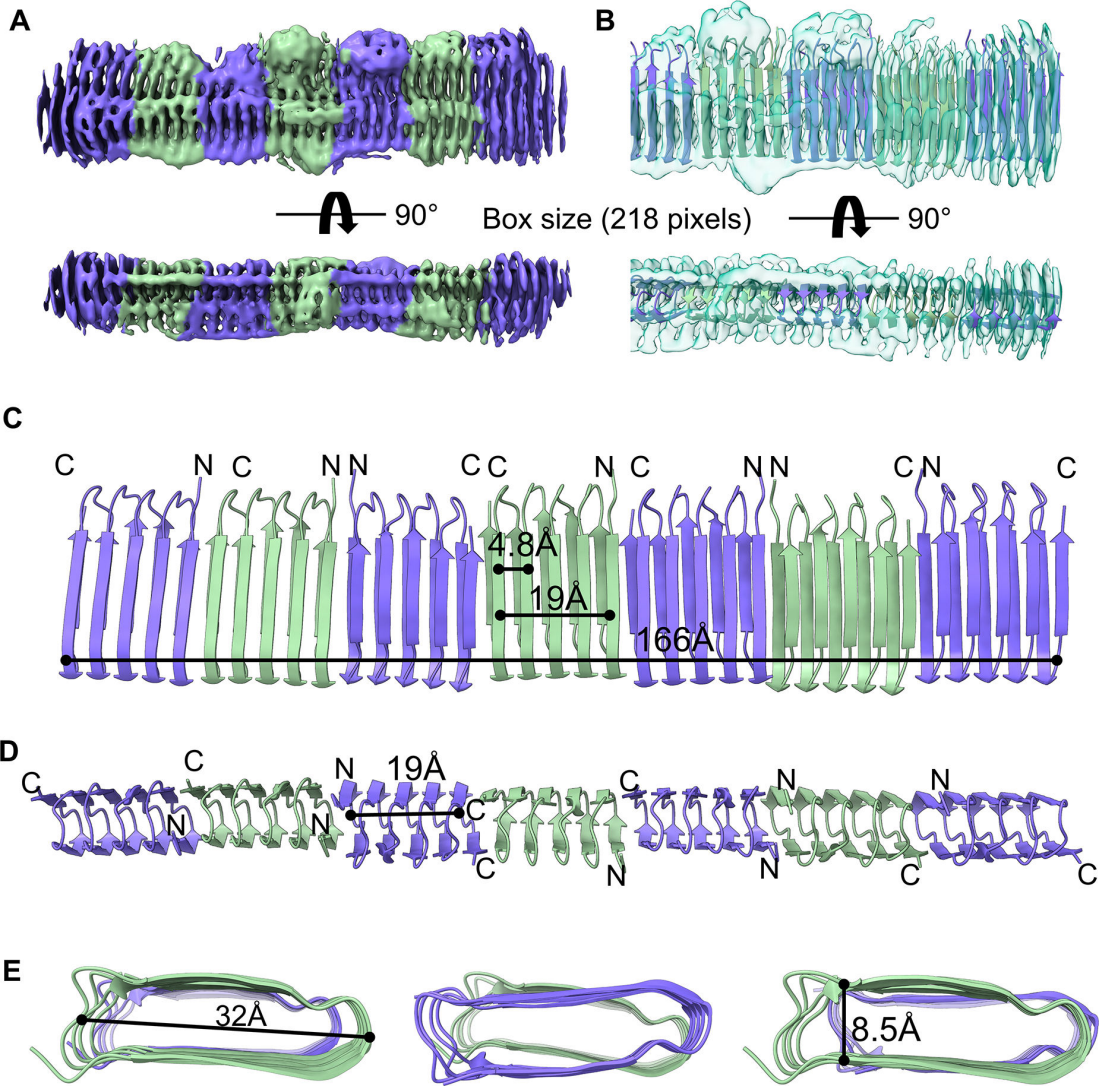
Cryo-EM structure of CsgA fibrils. Front and side views of the cryo-EM unsharpened map of CsgA fibrils (**A**) and with the structural model fitted (PDB: 8ENQ), presented as a ribbon cartoon (**B**). Front (**C**), side (**D**), and top (**E**) views of the CsgA fibril structural model and the beta-helical sizing of the fibril.

The AlphaFold predicted CsgA model was used as the initial model for docking. To discern the correct orientation, model assignment and adjustments were performed based on the additional density observed in the N22 region and the non-centrosymmetric properties of *E. coli* CsgA. The overall architecture of CsgA fibrils illustrated that seven and eight CsgA subunits could be assigned to the map of 218-pixel and 260-pixel box size, respectively. Fibrils comprising approximately 7–8 CsgA monomers appeared to exhibit high stability and uniformity (Fig. 4), potentially indicating a characteristic of CsgA aggregation. The CsgA fibrils model indicated that the adjacent strand distance and helix height are approximately 4.8 Å and 19.1 Å, respectively, exhibiting the closest agreement with those (4.8 Å and 19 Å) shown in the predicted CsgA model (29). In addition, our *E. coli* CsgA models also confirmed the previous prediction that the core-facing residues between beta strands are imperfect repeats, leading to the formation of steric zippers that are non-centrosymmetric (35). All three types of CsgA-CsgA interactions: head-to-head, head-to-tail, and tail-to-tail could be assigned to the current density map (Fig. 4). The curli repeat has a beta-solenoid structure with a single strand (2.4 Å) stagger between the two sheets. If the N22 imperfect half-repeat is not incorporated into the fibril core, then CsgA ends in a full repeat, with a 1-strand overhang on the N-terminus (start of R1) and on the C-terminus (end of R5). Given that R1 and R5 are interchangeable (38), these overhangs are expected to lead to degeneracy between head-head or head-tail connectivity, although the latter may be slightly preferred, based on docking results using RosettaDock (35). However, unambiguous determination of monomer connectivity was not possible here. Due to the limit of map quality as a result of preferred orientation, the real resolutions (around 4–6 Å) (Fig. S6) are lower than the reported values (3.6–3.8 Å) by cryoSPARC, and therefore, the detailed interactions between these molecules within a fibril need to be revealed by further higher-resolution structures.

### Cryo-EM density maps uncover two distinct spatial arrangements of CsgA fibrils

**Fig 5:**
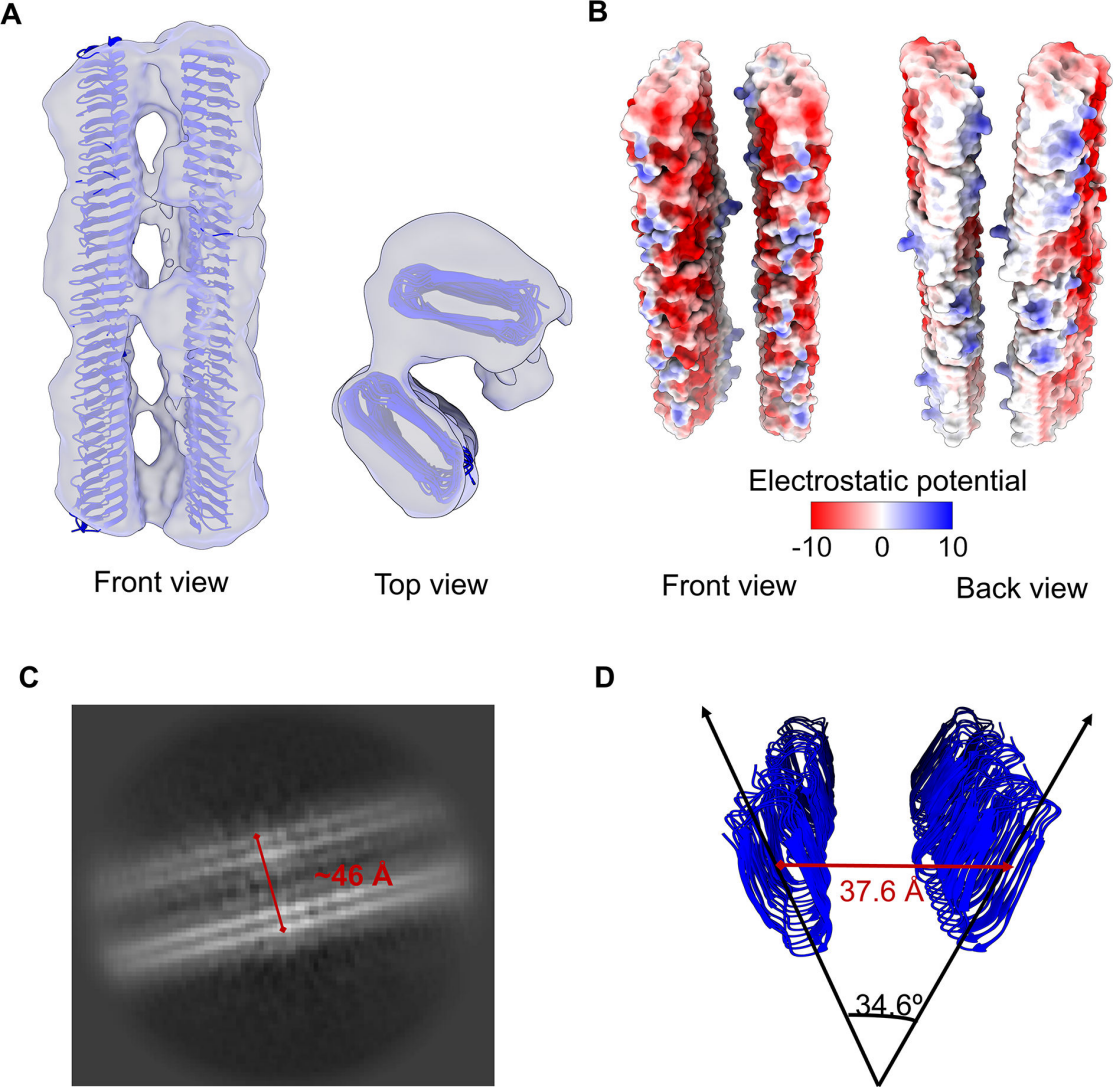
Predominant spatial organization of CsgA fibrils. (**A**) A 2-CsgA-fibril pair (front and top views). The CsgA model (PDB: 8ENQ) was docked into the class 3 cryo-EM map generated with a 260-pixel box size. (**B**) Electrostatic potential of a 2-CsgA-fibril pair uncovered the hydrophobic attraction between the side of two CsgA fibrils and the electrostatic repulsion (negative to negative charge) between the front of two CsgA fibrils. (**C**) A representative 2D class of 2-CsgA-fibril pair (side view). (**D**) The fibrils were tilted 34.6° relative to each other, and the distance between their centers was 37.6 Å. The angle and distance were measured in PyMoL.

Interestingly, we also discovered two major spatial arrangements of CsgA fibrils in class 2 (15.5%, 120,429 particles) and class 3 (25.5%, 198,126 particles) of cryo-EM density generated with a 260-pixel box size: a 3-CsgA-fibril bundle (Fig. S8) and a 2-CsgA-fibril pair (Fig. 5). Unlike the recent discovery in *P. korlensis* R15.5, where fibrils were found to be parallelly stacked in a planar array, *E. coli* CsgA fibrils in this structure exhibited a more complex spatial organization. Surprisingly, in the 2-CsgA-fibril pair, two protofilaments made contact through the sides at a specific angle of approximately 34° (Fig. 5D). This contact likely results from the attraction between uncharged residues on the sides of the fibrils through hydrophobic interactions, while there is repulsion between negatively charged residues on the front and back of the fibrils (see Fig. 5B). The distance between the centers of the two protofilaments was measured at 37.6 Å. This angle could explain the observed distance (46 Å) between two CsgA fibrils observed in the 2D class (as shown in Fig. 5C). Additionally, a less dominant but distinct arrangement, the 3-CsgA-fibril bundles (class 2, 15.5%), also formed at a specific angle (62°, 44°, 74°) and interacted through the sides of the protofilaments (Fig. S8). This may explain the occasional turns of CsgA fibril bundles observed in negative TEM images, despite the absence of helical twist morphology in a single fibril.

In summary, our study successfully optimized protein expression, post-fibrilization conditions, and image processing for *E. coli* CsgA fibrils. Using cryo-EM, we have shed the first light on the cryo-EM structure of *E. coli* CsgA fibrils, revealing that the amylogenic regions of CsgA adopt non-centrosymmetric beta-helical structures. Additionally, N22 appears to be flexible and partially interacts with the sides of the fibrils, forming an “ear-like” density. Furthermore, the beta-helical dimensions of CsgA closely match the previously predicted parameters. We also identified a unique helical thickness of *E. coli* CsgA by comparing it with *P. korlensis* R15.5. Notably, our study unveiled two distinct spatial arrangements of CsgA fibrils: 2-CsgA-fibril pairs and 3-CsgA-fibril bundles, in which individual fibrils interacted at a specific angle of ~34°. These findings provide crucial structural insights into CsgA fibrils for drug design, enhance our understanding of curli fibril organization in biofilms, and offer guidance for the precise design of curli-based biomaterials.

## MATERIALS AND METHODS

### Protein expression and purification

Recombinant CsgA_A63C/V140C was purified as described previously with some modifications (36, 38, 45). NEB 3016 ΔslyD cells harboring a pET11d vector encoding C-terminal His_6_-tagged CsgA_A63C/V140C were grown up to optical density at 600 nm (OD_600)_ ~0.8 in LB broth containing 100 µg/mL ampicillin at 37°C. The expression of CsgA_A63C/V140C monomer was induced with 0.5 mM IPTG at 37°C for 1–4 h or 28°C overnight to select the optimized induction time and temperature to achieve maximal yield. Cells were immediately harvested by centrifugation at 4,000 rpm for 20 min at 20°C and stored at −80°C prior to purification. Cell pellets corresponding to 750 mL of the induced cell culture were resuspended in 50 mL lysis buffer (8 M GdnHCl, 50 mM Kpi, pH 7.3), sonicated for 15 min, and shaken for 1 h at room temperature. The supernatant of the cell lysate was collected by centrifugation at 10,000 × *g* for 20 min at 20°C. After sonication (15 min) and filtration (0.22 µm), the supernatant was mixed with an equivalent volume of equilibration buffer (50 mM sodium phosphate, 300 mM sodium chloride, 6 M guanidine • HCl, 10 mM imidazole, pH 7.4) and purified using FPLC (AKTA Pure System) and a HisPur Cobalt column (Thermo Scientific). Protein was eluted with a non-denaturing elution buffer (50 mM sodium phosphate, 300 mM sodium chloride, 150 mM imidazole, pH 7.4), and every 1 mL fraction was examined by SDS-PAGE and western blotting. Fractions containing CsgA were pooled together. Then, a 30-kDa cutoff spin-filter (Amicon Ultra) was used to remove any potential aggregates, and the flow-through was collected and loaded onto a size exclusion column (Superdex 75 Increase 10/300) to further purify CsgA. Purified CsgA monomer solution (~0.5 mg/mL) was stored at 4°C less than 2 weeks prior to analysis.

### Monitoring the fibrilization kinetics of CsgA using thioflavin T (ThT)

*E. coli* CsgA fibrils were highly unstable and tended to form fibril bundles due to the lack of charge repulsion. To maximize the colloidal stability of CsgA fibrils for cryo-EM imaging and data processing, several fibril growth conditions (pH, salts, and additives) were screened in 96-well plates. Purified CsgA (40 µM) was diluted in HEPES at pH 7 or CAPS at pH 10.4 at different concentrations (10 mM and 50 mM) in the presence or absence of crystal screening additives (Ionic Liquid Screen Kit, Hampton Research, Aliso Viejo, CA, USA). Preliminary results revealed that the optimal temperature for CsgA fibril self-assembly was 25°C. In addition, due to the limited solubility and yield of CsgA monomer, 20 µM of CsgA was chosen for assembling fibrils. 20 µM ThT was mixed with CsgA monomer in the presence of 8 mM TCEP and incubated at 25°C for 14 h with or without continuous shaking (linear shake mode). The ThT signal was monitored using the BioTek Synergy Neo 2 (Agilent Technologies, Santa Clara, CA, USA) plate reader at an excitation and emission wavelength of 450 nm and 495 nm, respectively.

### Evaluating the morphology of CsgA fibrils using negative-staining TEM

CsgA fibrils were grown following the above-mentioned screening conditions in the absence of ThT for further characterization under negative stain TEM. Copper grids with a 300-mesh size, coated with a thin layer of continuous carbon (EM Sciences), underwent a glow-discharge treatment at 15 mA for 30 s. Next, 3 µL of CsgA fibrils (2 µM) was applied to these grids for a duration of 30 s. To stain the sample on the grid, 5 µL of uranyl formate solution (1% concentration, wt/vol) was employed for a 1-min period. Subsequently, the specimen was gently blotted from the side using filter paper and allowed to air-dry before imaging. Grids containing CsgA fibrils were imaged using a Tecnai Spirit Biotwin TEM microscope from FEI, equipped with a Gatan 4 K × 4 K CCD camera.

### Cryo-EM sample preparation and data acquisition

Two microliters of 3-([3-Cholamidopropyl]dimethylammonio)-2-hydroxy-1-propanesulfonate (CHAPSO) was added and mixed with 23 µL CsgA fibrils to get a final concentration of 8 mM immediately before grid preparation. Quantifoil R1.2/1.3 300 mesh Cu grid (EM Sciences) was glow-discharged at 15 mA for 60 s and a drop of 4 µL of fibrils was applied to the grid. The grid was then blotted for 4 s at 22°C and 100% humidity and vitrified by plunging into liquid ethane using a Vitrobot Mark IV (FEI). The cryo-EM data were acquired using a 300-keV Titan Krios microscope (FEI) equipped with a K3 direct electron detector with a Biocontinuum energy filter (Gatan) at the Hormel Institute, University of Minnesota. Automated data acquisition was carried out using Latitude-S (Gatan) in counting mode with a pixel size of 0.664 Å, a defocus ranging from −0.75 µm to −2.5 µm, a slit width of 20 eV, a dose rate of 25e−/Å^2^/s, and a total dose of 50e−/Å^2^.

### Image processing

Cryo-EM data were processed using cryoSPARC v3.3.2 (46), and the procedure is outlined in Fig. S4 and S5. In brief, beam-induced motion and mechanical drift were corrected with dose-weighting using the Patch motion correction (47), with data downsampled by 4/3 (0.885333 Å/pixel after downsampling), followed by determining the contrast transfer functions (CTFs) of the summed micrographs using Patch CTF estimation (48). Particles were then automatically picked using Blob picker with 40–300 Å particle diameters, and then junk particles were removed through multiple rounds of 2D classifications. Particles (1,231,430 or 776,966) in the good 2D classes were extracted using 218-pixel and 260-pixel box sizes and used for ab-initio reconstruction of four maps. The initial models were low-pass filtered to 20 Å and set as the starting reference for heterogeneous refinement (3D classification). Final particles (360,809 or 328,931) in the good class were subjected to homogeneous refinement, applying mask and non-uniform refinement successively, generating the final 3.62 Å map (218-pixel box size) and 3.79 Å map (260-pixel box size) for *E. coli* CsgA fibrils. Resolutions of the maps were determined by gold-standard Fourier shell correlation (FSC) at 0.143 between the two half-maps. Local resolution variation was estimated from the two half-maps by cryoSPARC v3.3.2. It is noteworthy to mention that the dip observed in the FSC curves at approximately 5 Å corresponds to the spacing between the windings of the beta helix in CsgA fibrils. Similar phenomena have been observed in other bacterial fibrils that adopt similar beta-helical structures (35, 49). Additionally, anisotropy in the cryo-EM map arises from the beta stacking along the filament, which is a common issue associated with amyloid fibrils (50, 51). This anisotropy can limit the quality of the density map, making it more challenging to interpret and extract detailed structural information from the amyloid fibrils.

### Model building and refinement

An initial CsgA monomer model, lacking the signal peptide, was generated using the online server AlphaFold and docked into the cryo-EM density map using Chimera (v1.17.3) (52) and COOT (53), along with manual real-space refinement and regularizing zone. The intact model was then refined using Phenix (54). In the real-space refinement, global minimization and local grid search were performed with the secondary structure, rotamer, and Ramachandran restraints applied throughout the entire refinement. The final models had good stereochemistry by evaluation in MolProbity (55). Due to the anisotropy in the cryo-EM map arising from the beta stacking and the preferred orientation issue, the actual resolutions of the maps are around 4–6 Å, and therefore, side chains of the models are not well defined. Three-dimensional FSCs for assessing directional resolution anisotropy were calculated using the online server (https://3dfsc.salk.edu) (56). The map sphericity values of two cryo-EM maps in this study are lower than 0.8, suggesting the preferred orientation problem. However, resolving preferred orientation is challenging due to the inherent structural characteristics of elongated, rod-like CsgA fibrils. The statistics of cryo-EM data collection, 3D reconstruction, and model refinement are shown in Table S2. All figures were generated using UCSF ChimeraX (v1.4) (57).

## Data Availability

Atomic coordinates of CsgA amyloid fibril structures have been deposited in the PDB with accession number 8ENQ (218-pixel box size) and 8ENR (260-pixel box size). The cryo-EM density maps have been deposited in the Electron Microscopy Data Bank with accession number EMD-28276 (218-pixel box size) and EMD-28277 (260-pixel box size).

## SUPPLEMENTAL MATERIAL

Supplemental material mbio.00419-24-s0001.pdf Fig. S1 to S8; Tables S1 and S2.

ASM does not own the copyrights to Supplemental Material that may be linked to, or accessed through, an article. The authors have granted ASM a non-exclusive, world-wide license to publish the Supplemental Material files. Please contact the corresponding author directly for reuse.
