# Supplementary material for: Structural insight into Escherichia coli CsgA amyloid fibril assembly
Source: mBio. 2024 Mar 19;15(4):e00419-24. doi: 10.1128/mbio.00419-24 (PMC11005368; doi:10.1128/mbio.00419-24)
Supplement: Supplemental material — Fig. S1 to S8; Tables S1 and S2. [file mbio.00419-24-s0001.pdf]

Supplementary Information to

Structural insight into *Escherichia coli* CsgA amyloid fibril assembly

Author List: Fan Bu[1,2,3#], Derek R. Dee[2#], Bin Liu[1#]

Affiliations:

[1]Section of Transcription & Gene Regulation, The Hormel Institute, University of Minnesota, Austin, MN, USA

[2]Faculty of Land and Food Systems, The University of British Columbia, Vancouver, BC, Canada

[3] Department of Pharmacology, University of Minnesota Medical School, Minneapolis, MN, USA

[#]Correspondence:

bu007102@umn.edu (F.B.); derek.dee@ubc.ca (D.D.); liu00794@umn.edu (B.L.)

Contents:

Supplementary Figures: 1-8

Supplementary Tables: 1-2

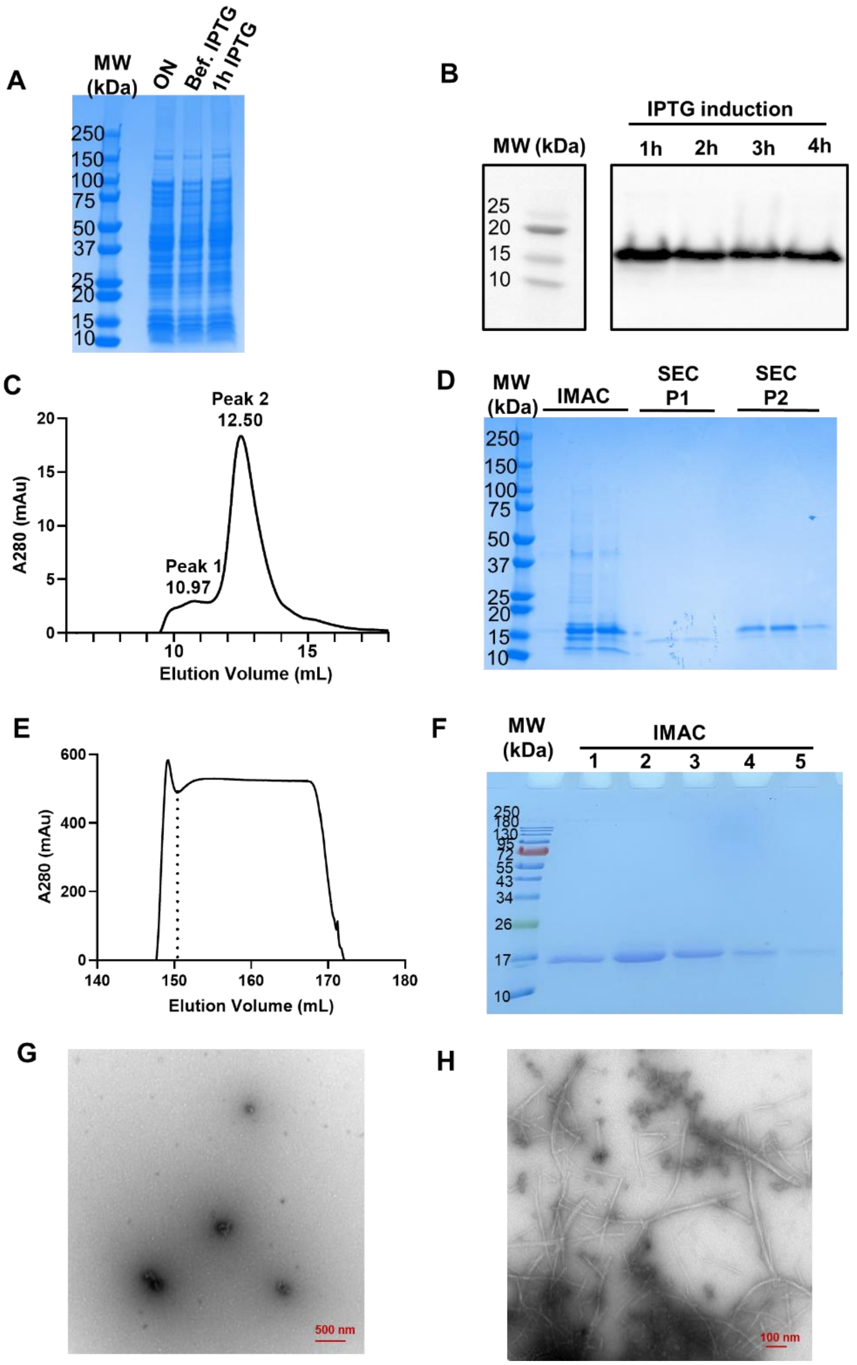

Supplementary Figure 1: Expression and purification of CsgA in the absence of reducing agent. **(A)** No CsgA band (~15 kDa) was apparent on SDS-PAGE after IPTG induction. **(B)**The expression of CsgA was confirmed by western blot. **(C)** Size exclusion chromatogram of CsgA purified from 1L of LB and passed through a nickel column and a 30-kDa spin filter. **(D)** SDS-PAGE revealed that the eluates collected from the nickel column contained impurities and were successfully purified using size exclusion chromatography. **(E, F)** One-step purification of CsgA from 500 ml TB using cobalt column to obtain pure CsgA. (G) TEM image of CsgA without reducing agent. (H) TEM image of CsgA 40 h after adding TCEP.

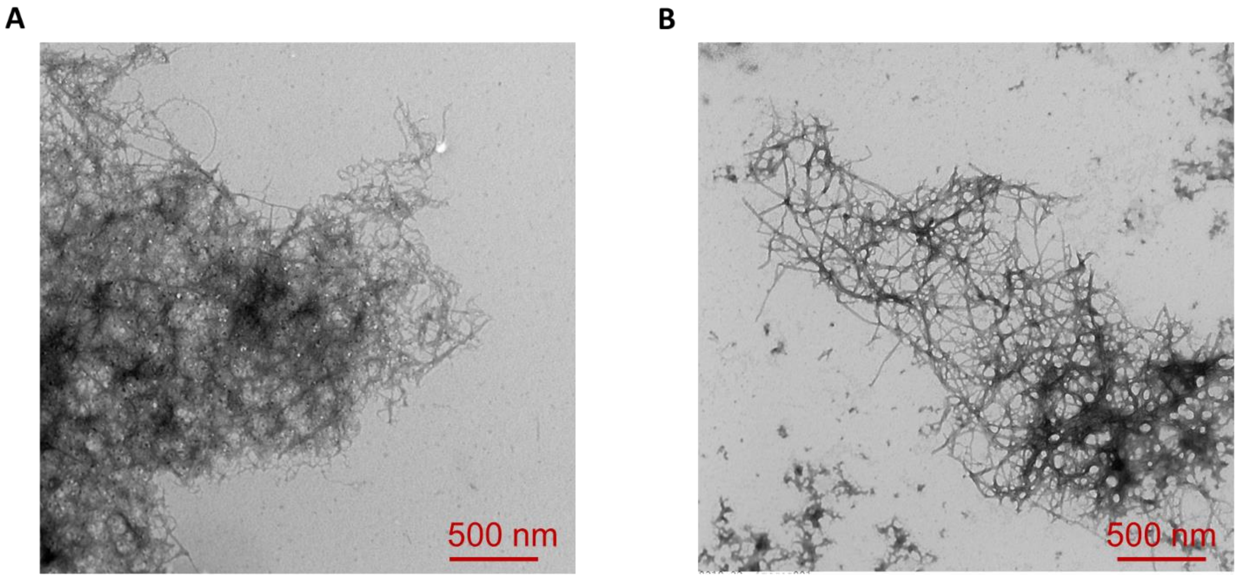

Supplementary Figure 2: Negative staining TEM of CsgA fibrils obtained in PBS **(A)** and Tris-HCl, pH 7.2 **(B)**.

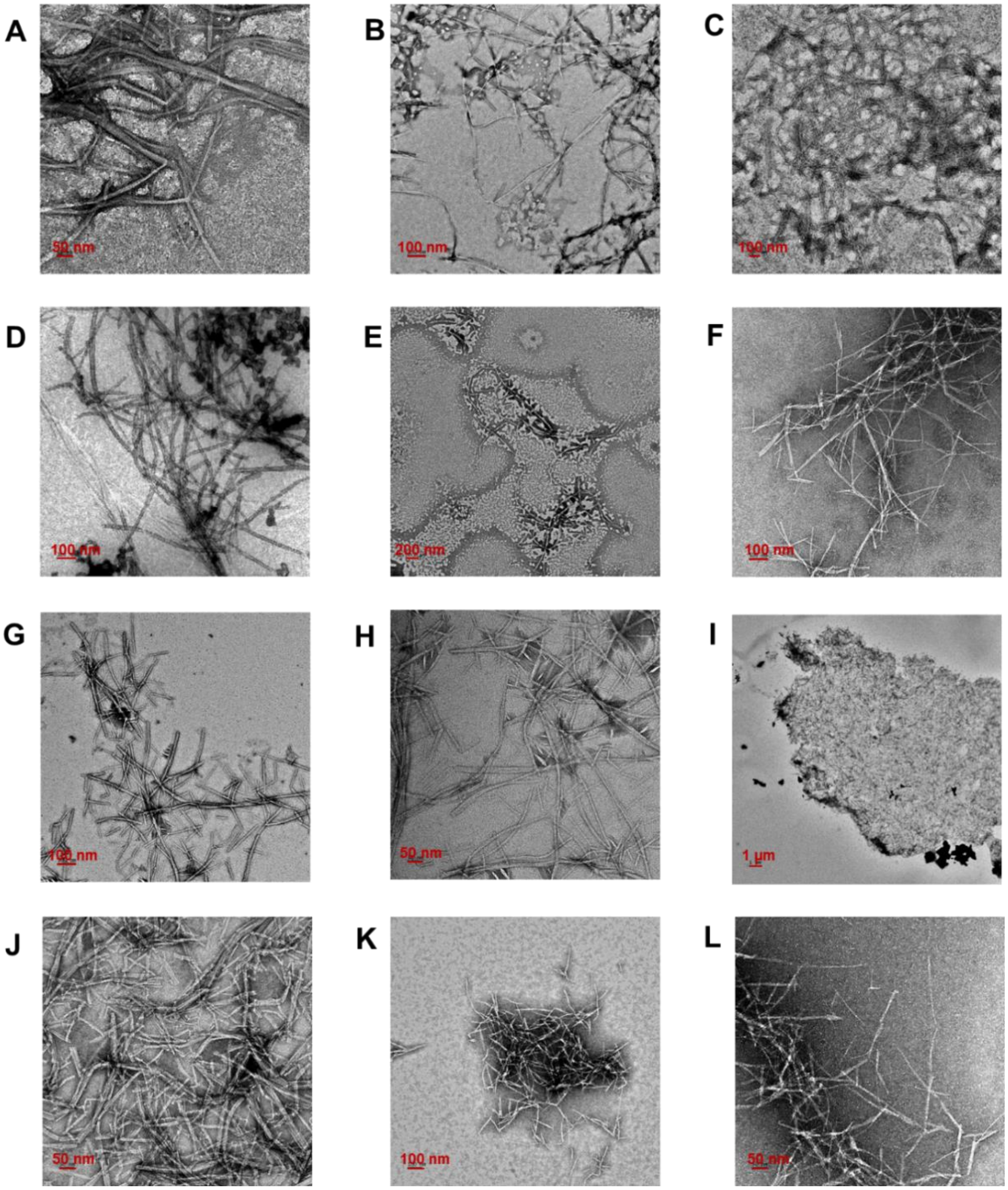

Supplementary Figure 3: Negative staining TEM revealed apparently distinct morphologies for CsgA fibrils obtained under various growth and post-fibrillation conditions. **(A-F)** CsgA fibrils were grown in the presence of different crystallization buffers (1-Butylpyridinium chloride, Triisobutylmethylphosphonium tosylate, Tetrabutylphosphonium bromide, Tetraethylammonium bromide, Benzyltriethylammonium chloride, or 2-Hydroxyethylammonium formate). **(G, H)** CsgA fibrils, formed at 10 mM HEPES buffer (pH 7) +0.01% Tween 20, without shaking, were heated at 70 ºC for 30 min. **(I, J)** CsgA fibrils, formed at the same condition, were dialyzed against MilliQ water. **(K, L)** CsgA fibrils at the same condition, were sonicated 20 s, three times.

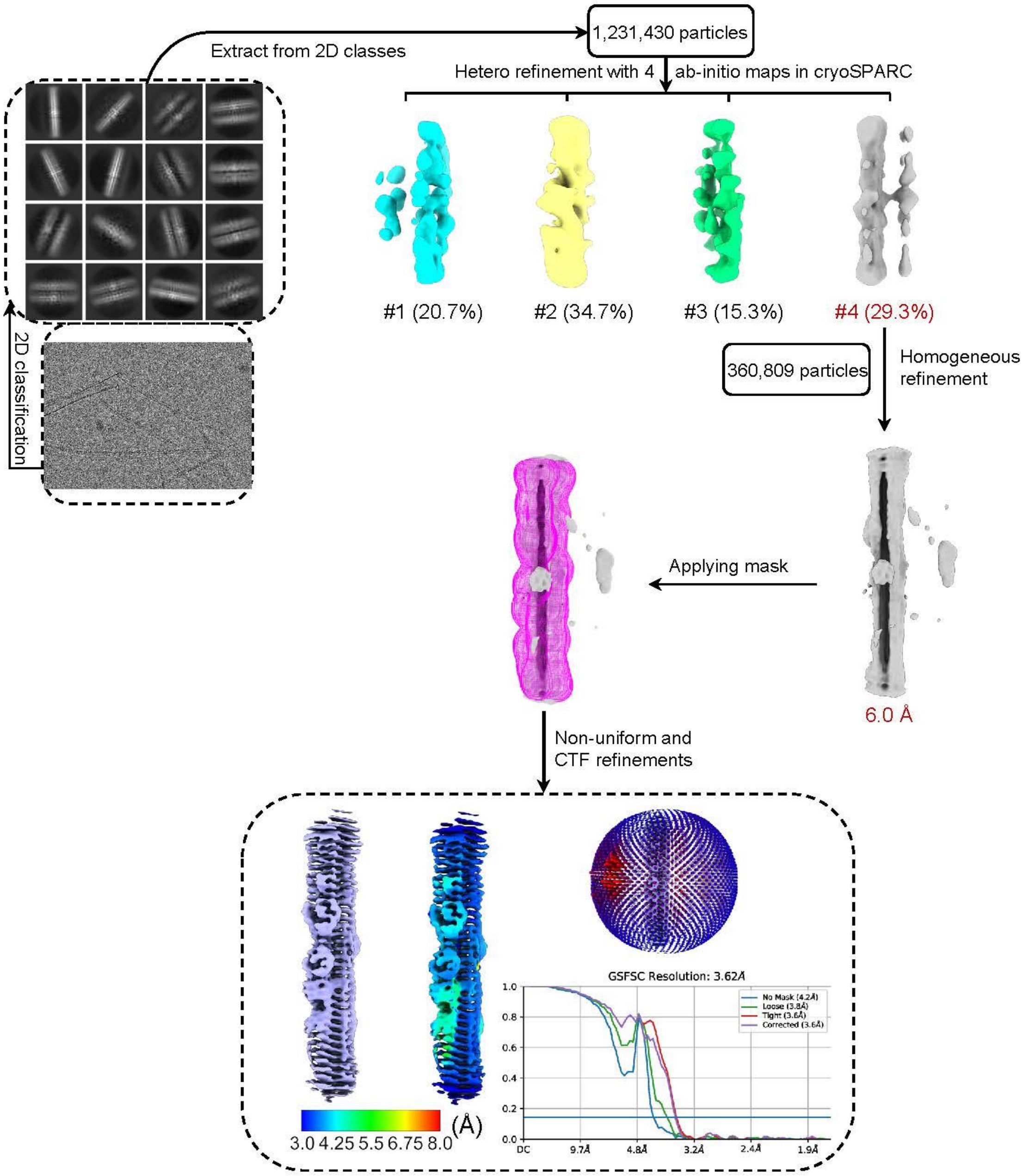

Supplementary Figure 4: Flow chart of cryo-EM image processing of CsgA fibrils with a 218-pixel box size. Representative raw cryo-EM image and 2D classes are presented. 3D refinement using all the particles in good 3D classes generated a 6.0 Å map. Further masked 3D classification generated one fibril conformation. Angular distribution plot is displayed. The final maps, half-map FSC curves and accompanying local resolution illustrations are enclosed in the dashed black box. The dip in the FSC curves at around 4.8 Å corresponds to the beta helix winding spacing in CsgA fibrils, similar to observations in other bacterial fibrils with comparable beta helical structures (1, 2). Anisotropy in the cryo-EM map is also common due to beta stacking in amyloid fibrils.

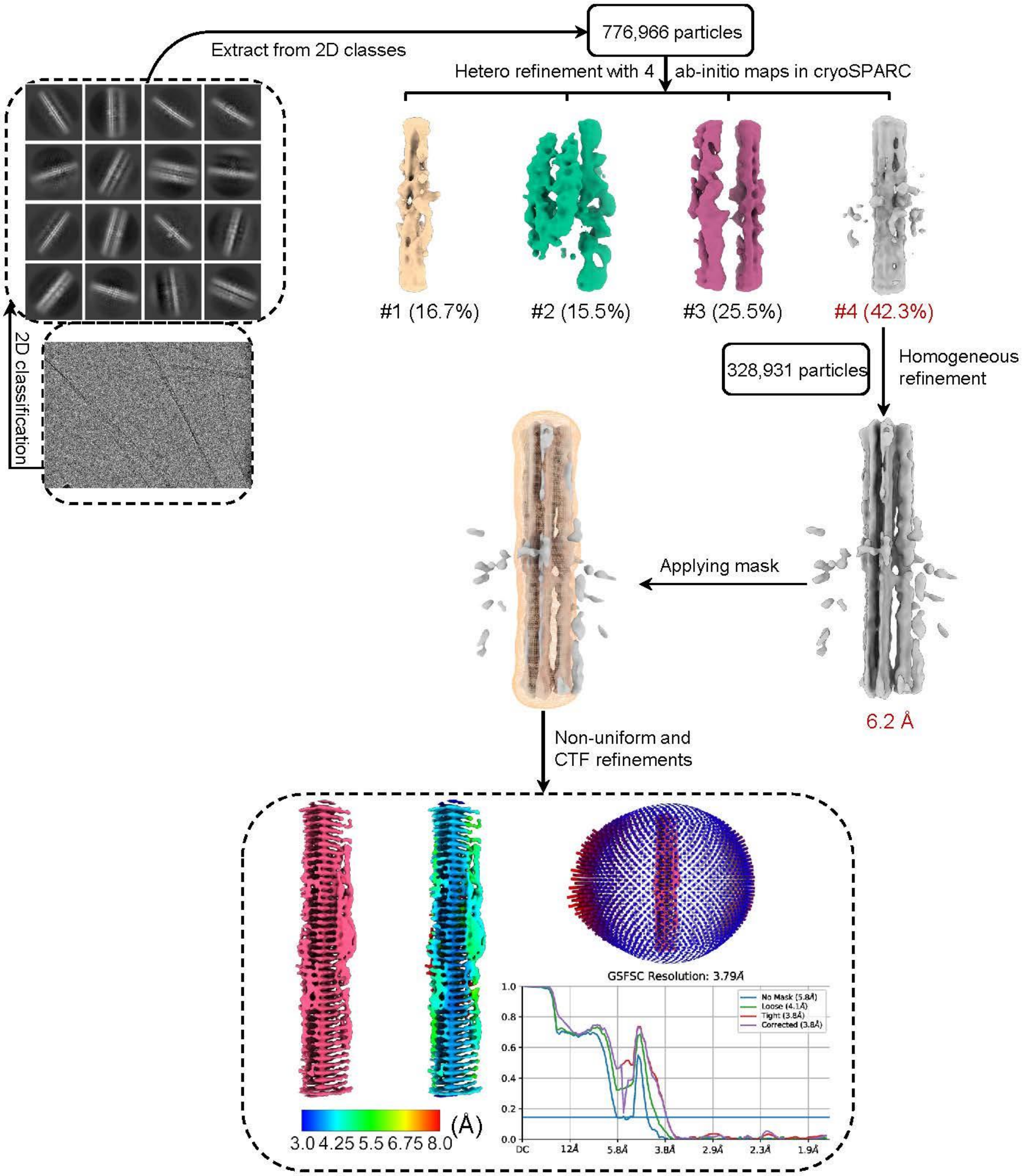

Supplementary Figure 5: Flow chart of cryo-EM image of CsgA fibrils with a 260-pixel box size. Representative raw cryo-EM image and 2D classes are presented. 3D refinement using all the particles in good 3D classes generated a 6.2 Å map. Further masked 3D classification generated one fibril conformation. Angular distribution plot is displayed. The final maps, half-map FSC curves and accompanying local resolution illustrations are enclosed in the dashed black box. The dip in the FSC curves at around 4.8 Å corresponds to the beta helix winding spacing in CsgA fibrils, similar to observations in other bacterial fibrils with comparable beta helical structures (1, 2). Anisotropy in the cryo-EM map is also common due to beta stacking in amyloid fibrils.

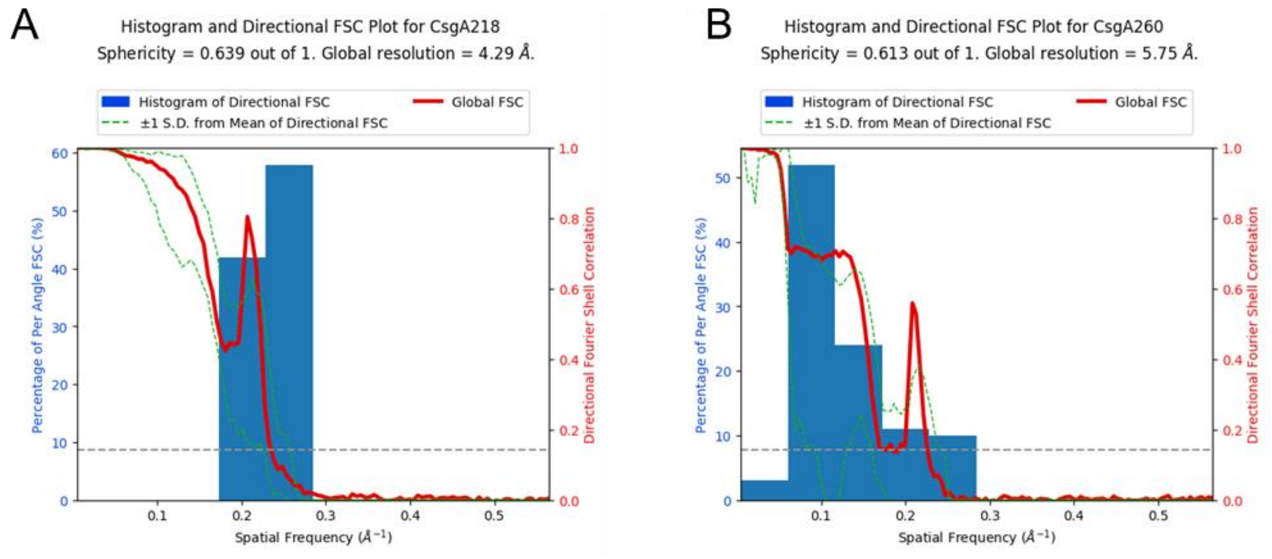

Supplementary Figure 6: Histogram and directional FSC plots of the final 3D reconstruction of CsgA amyloid fibril with a 218-pixel box size **(A)** and a 260-pixel box size **(B)**. The sphericity values of both maps are indicated.

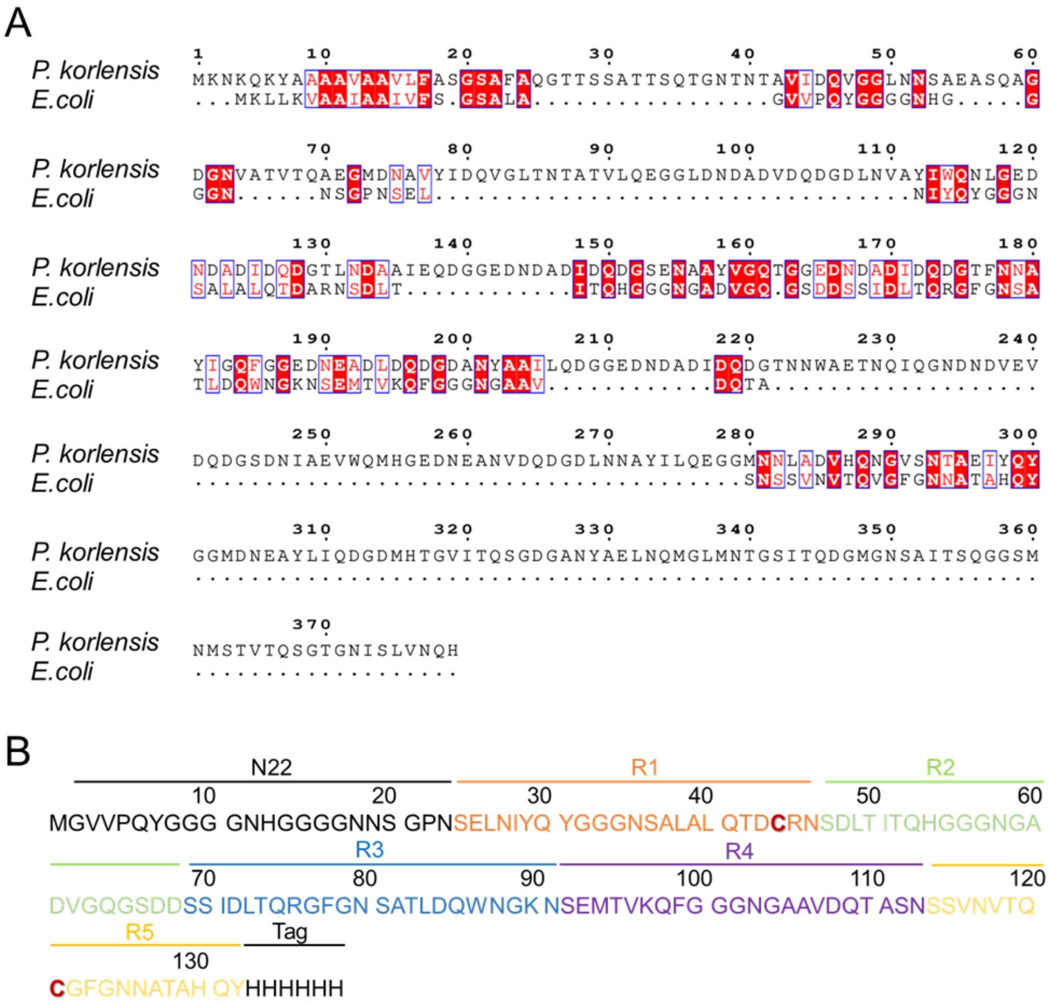

Supplementary Figure 7: (A) Sequence alignment (Clustal Omega) of CsgA from *P. korlensis* and *E. coli* shows distinct differences. (B) Sequence information for CsgA_A63C/V140C.

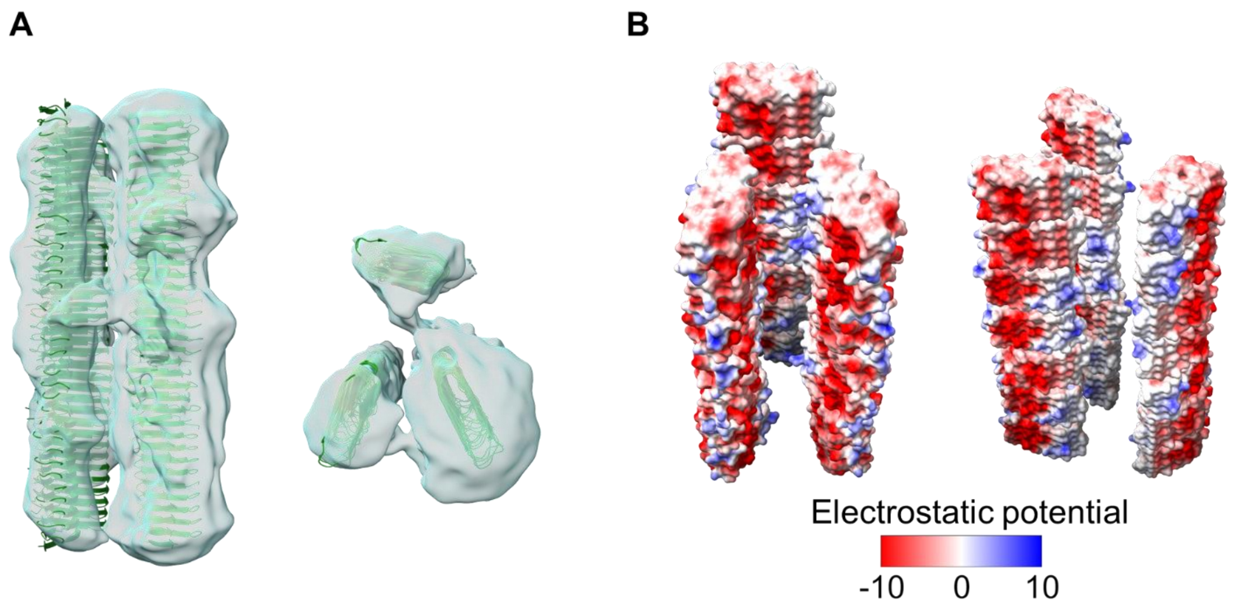

Supplementary Figure 8: Class #2 (Supplementary Figure 5) of cryo-EM map generated with a 260-pixel box size uncovered a spatial organization among three CsgA fibrils. **(A)** 3-CsgA-fibril bundle (front and top views). **(B)** Electrostatic potential of 3-CsgA-fibril bundle indicated the electrostatic repulsion likely responsible for the de-bundling of CsgA fibrils.

Supplementary Table 1: Cryo-EM data collection, refinement and validation statistics.

	E. coli CsgA fibril<br>218-pixel box size<br>(EMDB-28276)<br>(PDB 8ENQ)	*E. coli* CsgA fibril<br>260-pixel box size<br>(EMDB-28277)<br>(PDB 8ENR)
**Data collection and processing**		
Magnification	96,000	96,000
Voltage (kV)	300	300
Electron exposure (e–/Å^2)	40	40
Defocus range (μm)	-1.0 ~ -2.4	-1.0 ~ -2.4
Pixel size (Å)	0.89	0.89
Symmetry imposed	C1	C1
Initial particle images (no.)	1,231,430	776,966
Final particle images (no.)	360,809	328,931
Map resolution (Å)	3.6	3.8
FSC threshold	0.143	0.143
Map resolution range (Å)	3.0–8.0	3.0–8.0
**Refinement**		
Map sharpening *B* factor (Å^2)	-182.1	-186.9
Model composition		
Non-hydrogen atoms	5600	6400
Protein residues	777	888
B factors (Å^2)		
Protein	49.04	106.48
R.m.s. deviations		
Bond lengths (Å)	0.004	0.004
Bond angles (°)	0.766	0.800
**Validation**		
MolProbity score	1.67	1.73
Clashscore	14.48	17.12
Poor rotamers (%)	0	0.89
Ramachandran plot		
Favored (%)	99.74	99.20
Allowed (%)	0.26	0.80
Disallowed (%)	0.00	0.00

Supplementary Table 2: Comparison of beta-helical sizing of CsgA obtained from experimental data and molecular simulation.

CsgA	Adjacent strand distance (Å)	Helix height (Å)	Helix thickness (Å)	Helix width (Å)
**Experimental Data**				
CsgA (*Escherichia coli*, cryo-EM)	4.8±0.1	19.1±0.1	8.6±0.2	32.7±0.7
R15.5 (*Pontibacter* korlensis, cryo-EM) (2)	~4.8	N/A	~20	~37
CsgA (*Escherichia coli*, x-ray diffraction) (3)	~4.7	N/A	9	N/A
**Molecular simulation**				
CsgA (*Escherichia coli*) (4)	4.8	~19	~9	~32
CsgA (*Escherichia coli*) (5, 6)	4.9 ± 0.4	19.2 ± 0.9	9.6 ± 1.7	28.8 ± 2.0

References:

1. Deng X, Gonzalez Llamazares A, Wagstaff JM, Hale VL, Cannone G, McLaughlin SH, Kureisaite-Ciziene D, Löwe J. 2019. The structure of bactofilin filaments reveals their mode of membrane binding and lack of polarity. Nature microbiology 4:2357-2368.
2. Sleutel M, Pradhan B, Volkov AN, Remaut H. 2023. Structural analysis and architectural principles of the bacterial amyloid curli. Nature Communications 14:2822.
3. Shewmaker F, McGlinchey RP, Thurber KR, McPhie P, Dyda F, Tycko R, Wickner RB. 2009. The functional curli amyloid is not based on in-register parallel β-sheet structure. Journal of Biological Chemistry 284:25065-25076.
4. Tian P, Boomsma W, Wang Y, Otzen DE, Jensen MH, Lindorff-Larsen K. 2015. Structure of a functional amyloid protein subunit computed using sequence variation. Journal of the American Chemical Society 137:22-25.
5. DeBenedictis E, Ma D, Keten S. 2017. Structural predictions for curli amyloid fibril subunits CsgA and CsgB. RSC advances 7:48102-48112.
6. Dunbar M, DeBenedictis E, Keten S. 2019. Dimerization energetics of curli fiber subunits CsgA and CsgB. npj Computational Materials 5:27.
